# The Relationship between MACC1/c-Met/Cyclin D1 Axis Expression and Prognosis in ESCC

**DOI:** 10.1155/2022/9651503

**Published:** 2022-02-22

**Authors:** Yan Shi, Meng-Yan Li, Hui Wang, Chao Li, Wen-Ying Liu, Yong-Mei Gao, Bo Wang, Jia-Wei Song, Yu-Qing Ma

**Affiliations:** ^1^Department of Pathology, The First Affiliated Hospital, Xinjiang Medical University, Urumqi, Xinjiang, China; ^2^Xinjiang Medical University, Urumqi, Xinjiang, China; ^3^Department of RICU, The First Affiliated Hospital, Xinjiang Medical University, Urumqi, Xinjiang, China; ^4^Xi'an Medical University, Xi'an, Shaanxi, China

## Abstract

**Background:**

Esophageal cancer is one of the most common malignant tumors of the digestive system, with high incidence and mortality.

**Methods:**

Immunohistochemical method was used to detect the expression of MACC1, c-Met, and cyclin D1 in ESCC and its adjacent tissues. Statistical analysis was done by SPSS 23.0.

**Results:**

The high expression of MACC1 and cyclin D1 was significantly correlated with tumor size. High c-Met expression was associated with patient ethnicity. MACC1 expression was positively correlated with both c-Met and cyclin D1. c-Met expression was also positively correlated with cyclin D1. Patients with high expression of MACC1 and c-Met had worse OS; patients with high c-Met expression also had worse PFS.

**Conclusion:**

MACC1, c-Met, and cyclin D1 proteins are closely related to the occurrence and development of esophageal squamous cell carcinoma. MACC1 may affect the prognosis of ESCC by regulating the expression of the c-Met/cyclin D1 axis.

## 1. Introduction

Esophageal cancer is one of the most common malignant tumors in the world, with high mortality and poor prognosis [1]. EAC is more common in western countries, while ESCC is dominant in China [2]. The number of male ESCC patients in China is far higher than that of female patients [3], and EC in China has obvious regional specificity in distribution, with a significantly high incidence in some areas of Xinjiang [4]. The exact cause of esophageal cancer is not known. However, it is related to living conditions, poor dietary habits, lack of nutrients, virus infection, and genetic susceptibility [5, 6], and there is familial aggregation of the disease without obvious inducement.

Under normal physiological conditions, HGF/c-Met can mediate embryonic development, cell proliferation, injured tissue repair, and neuromuscular formation [7]. A large number of studies have shown that the overactivation of c-Met may initiate the transformation of normal cells to tumor cells and further promote the occurrence of subsequent events such as invasion, metastasis, and diffusion [8]. c-Met is the encoding HGF receptor gene, located in 7q21-q31 on human chromosome 7; is a transmembrane receptor protein tyrosine kinase; and is expressed mainly in the epithelial tissue [9]. A typical marker of cell carcinogenesis is the occurrence of EMT, in which c-Met is believed to play a key driving role [10].

MACC1 is a biomarker newly discovered by Stein et al. in 2009 that can predict colorectal cancer metastasis and patient survival [11]. MACC1 is located on human chromosome 7p21.1. It contains 7 exons, encodes 852 amino acid residues, and contains 4 domains: SH3, ZU5, and 2 hydroxyl-terminated dead domains [12]. Recent studies have shown that MACC1 is a key regulatory factor in the HGF/c-Met signaling pathway and is a major target for tumor invasion and metastasis [13, 14]. MACC1 can bind to the c-Met promoter and enhance the proliferation of osteosarcoma cells and vascular endothelial cells through the HGF/c-Met signaling pathway [13]. MACC1 accelerated the activation of the HGF/c-Met/PI3K/AKT pathway and phosphorylated BAD, caspase 9, and FKHRL1, ultimately preventing hepatocellular carcinoma nuclear translocation and promoting apoptotic function [15].

Cyclin D1, is a protein encoded by the human CCND1 gene, with 5 exons and a full length of about 15 Kb, and is the smallest cyclin. Cyclin D1 is the regulator of cyclin-dependent kinase CDKs, whose main function is to promote cell proliferation [16, 17]. Cyclin D1 expression was significantly higher in high-grade invasive urothelial carcinoma than in low-grade noninvasive tumors (*P* < 0.05); its expression was found to be significant predictive factors of high-grade tumors [18]. Ramos-García et al. found that cyclin D1 was overexpressed in both oral squamous cell carcinoma and adjacent nonneoplastic epithelium and was positively expressed in the basal and parabasal nuclei of normal squamous epithelium. Ramos-García et al. [19] found that cyclin D1 overexpression was related to the mechanism of lip carcinogenesis and its asymmetric proliferation pattern by immunohistochemical methods [20]. In addition, a recent meta-analysis found that cyclin D1 overexpression was significantly associated with the malignant progression of head and neck squamous cell carcinoma, especially for the malignant risk of oral PMDs [21].

The purpose of this study was to preliminarily analyze the expressions of MACC1, c-Met, and cyclin D1 in esophageal squamous cell carcinoma and their relationship with clinicopathological parameters, as well as the effects of the expressions of the three proteins on the prognosis of ESCC.

## 2. Materials and Method

### 2.1. Patients and Tissue Samples

All patients gave informed consent before sample collection, and this study was approved by the Ethics Committee of the First Affiliated Hospital of Xinjiang Medical University (20180223-08). 172 ESCC paraffin-embedded samples and paired adjacent noncancerous tissues between January 2008 and June 2018 were collected and fabricated into tissue chips. Follow-up was conducted by inquiring medical records and telephone follow-up until July 2020.

Inclusion criteria were as follows: none of the patients received any preoperative treatment; esophageal primary tumor, not metastatic cancer of other sites; there were no other complications or organ tumors; and as a control group, normal epithelial tissue was at least 5 cm from the edge of the ESCC tissue. Exclusion criteria were as follows: esophageal adenocarcinoma; patients who had received radiotherapy or chemotherapy before surgery; tumor metastasis to the esophagus; and other ethnic groups: Uygur, Mongolian, etc. The following information was recorded for each patient: age, gender, ethnicity, tumor location, tumor size, degree of differentiation, TNM staging, lymph node status, vascular invasion, nerve invasion, and progression of disease.

All specimens underwent HE staining and sliced after placement in paraffin; then, all specimens were taken from patients who had been diagnosed and verified by a pathologist.

### 2.2. Antibodies and Reagents

The main antibodies and reagents were as follows: MACC1, c-Met, and cyclin D1. Other reagents are endogenous peroxidase blocker, normal goat serum working solution for sealant, biotin-labeled goat anti-rabbit IgG, horseradish enzyme-labeled streptomycin working solution, and 2-amino-benzidine (DAB), purchased from Zhongshan Jinqiao Company (Beijing, China).

### 2.3. Immunohistochemistry

The tissue chips are heated in an oven at 65 degrees Celsius for 45 minutes to soften the wax coating on the tissue chips. Tissues were fixed in 10% formalin, sectioned at 5 *μ*m, subsequently deparaffinized in xylene, and rehydrated in 100%, 95%, 80%, and 70% ethanol. All tissues were blocked with hydrogen peroxide for 10 min and heated in a microwave for antigen retrieval. After blocking with 1% goat serum, the sections were incubated with primary antibodies MACC1 (Bioss, bs-4293R, China, 1 : 100), c-Met (Bioss, bs-0668R, China, 1 : 300), and cyclin D1 (Dako, IS08330-2, Denmark, 1 : 500) for 90 min at 37°C.

After washing 3 times with PBS, 3 min each time, the sections were added dropwise with a secondary antibody (goat anti-rabbit IgG, SP-9001, ZSGB, China) and incubated at room temperature for 15 min. Afterwards, they were washed with PBS solution for 3 times, the unbound secondary antibody was thoroughly washed off, and DAB staining was performed. When the tissue is brown with the naked eye, it is rinsed in clean water. It was placed in hematoxylin solution for staining for 20 seconds, immersed in xylene solution for differentiation, and then dehydrated with gradient ethanol (70%, 80%, and 95%, absolute ethanol, concentration from low to high). Sections were air-dried in a fume hood and mounted dropwise with neutral resin gel.

The criteria for interpretation of immunohistochemical results are as follows: for MACC1 and c-Met, “0” for no color, “1” for light yellow, “2” for yellow, and “3” for brown. The percentage of positive cells was calculated under the view, and scoring was performed according to the following standards: ≤5%, a score of “0”; 6–25%, a score of “1”; 26–50%, a score of “2”; and 51–100%, a score of “3.” The final score was obtained by multiplying the average staining intensity of each slice by the average percentage of positive cells, with 0–4 score for negative (−) and 5–9 for positive (+) [21]. Immunoreactivity to cyclin D1 was “low” if nuclear staining of tumor cells was <20% (negative expression) and “high” if ≥20% (positive expression) [22].

### 2.4. Statistical Analysis

The characteristics of the ESCC patients were compared using the *χ*^2^ test. Overall survival (OS) and progression-free survival (PFS) were assessed using the Kaplan–Meier method and the log-rank test. Multivariate analysis was carried out using the Cox proportional hazard regression model. *P* < 0.05 was considered statistically significant.

## 3. Result

### 3.1. Clinicopathological Characteristics

The demographic and pathological characteristics of 172 patients with esophageal squamous cell carcinoma included in the study are summarized in Table 1. The median age at diagnosis was 62.5 years, and the mean age was 61.8 years (32-83 years). The majority of patients were male (118/68.6%). Patients were followed for a mean of 34 months (2-108 months). 67 cases (39.0%) died during the follow-up period. Among the 172 patients, 62 patients (36.0%) underwent radical surgery and postoperative chemoradiotherapy, and 114 patients (64.0%) underwent radical surgery.

### 3.2. Expression of MACC1, c-Met, and cyclin D1 in ESCC and Their Relationship with Clinicopathological Parameters

**Figure 1:**
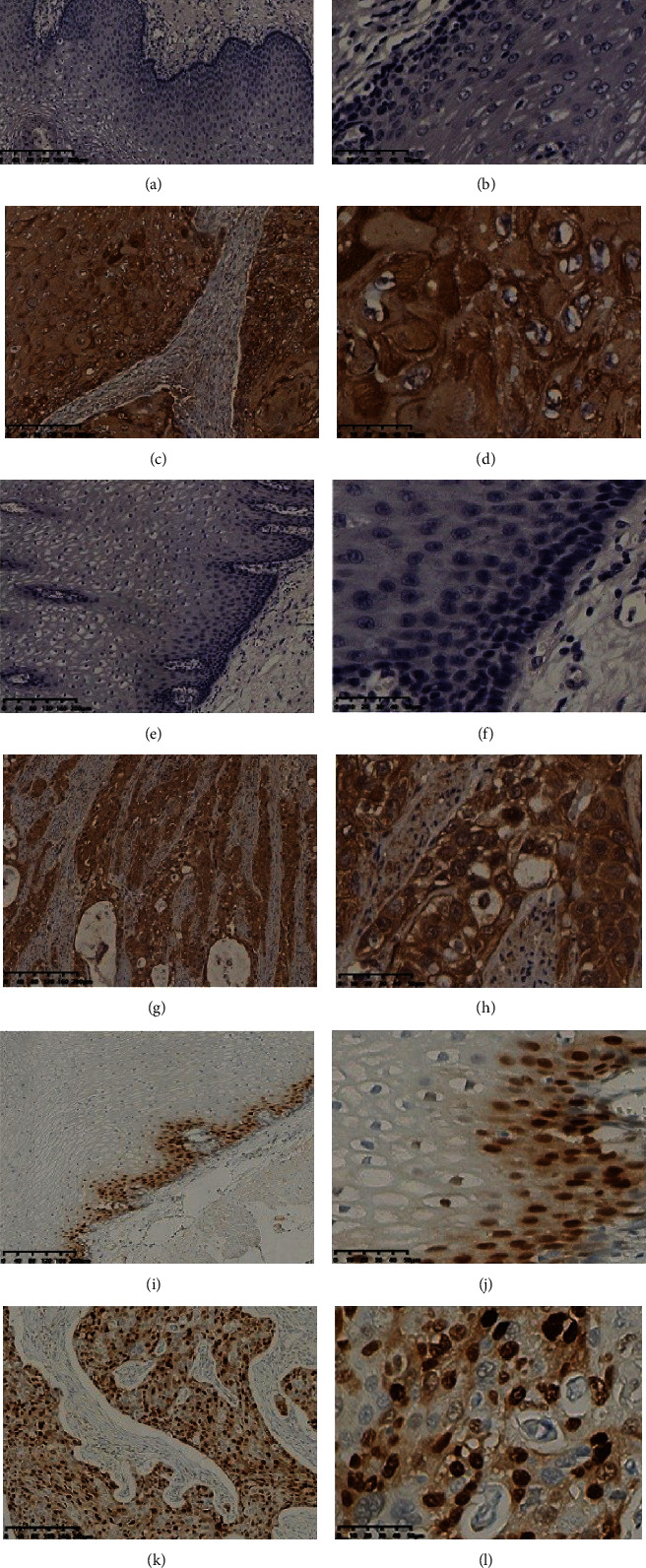
IHC staining of MACC1, c-Met, and cyclin D1 in ESCC tissues and normal esophageal mucosa. Notes: (a) MACC1-negative expression in normal esophageal tissues (×10); (b) MACC1-negative expression in normal esophageal tissues (×40); (c) MACC1-positive localized in cytoplasm expression in ESCC (×10); (d) MACC1-positive expression in ESCC (×40); (e) c-Met-negative expression in normal esophageal tissues (×10); (f) c-Met-negative expression in normal esophageal tissues (×40); (g) c-Met-positive expression localized in the cell cytoplasm and cell nucleus in ESCC (×10); (h) c-Met-positive expression in ESCC (×40); (i) cyclin D1 expression in mucosal basal lamina cells of normal esophageal tissues (×10); (j) cyclin D1-negative expression in normal esophageal tissues (×40); (k) cyclin D1-positive localized in nucleus expression in ESCC (×10); (l) cyclin D1-positive expression in ESCC (×40). Abbreviations: ESCC: esophageal squamous cell carcinoma; IHC: immunohistochemistry.

The expression of MACC1 in ESCC is shown in Figures 1(a)–1(d). MACC1 was positive in the cytoplasm of esophageal squamous cells and negative in normal esophageal mucosa. A total of 172 patients with ESCC were included in this study, among whom 80 (46.5%) were MACC1 negative and 92 (53.5%) were MACC1 positive. Statistical analysis showed that the high expression of MACC1 was related to tumor size (*P* = 0.02, *P* < 0.05). As shown in Table 2, positive MACC1 expression was associated with tumor size in Han patients with ESCC (*P* = 0.03, *P* < 0.05) and invasive depth (*P* = 0.03*P* < 0.05) which were significantly associated.

The expression of c-Met in ESCC and its relationship with clinicopathological parameters were investigated. As is shown in Figures 1(e)–1(h), c-Met-positive signals are brown and yellow in the cytoplasm and nucleus of esophageal carcinoma cells. In normal tissues adjacent to the cancer, c-Met expression was negative. All tumor specimens were divided into the c-Met-positive group (98 cases, 57.0%) and the c-Met-negative group (74 cases, 43.0%). c-Met expression and clinicopathological parameters are shown in Table 3. Statistical analysis showed that the positive expression of c-Met was related to ethnicity (*P* = 0.01, *P* < 0.05). However, c-Met-positive expression was associated with age (*P* = 0.11), gender (*P* = 0.68), tumor location (*P* = 0.50), tumor size (*P* = 0.17), degree of differentiation (*P* = 0.12), lymph node metastasis (*P* = 0.98), invasive depth (*P* = 0.87), AJCC stage (*P* = 0.25), vascular invasion (*P* = 0.60), nerve invasion (*P* = 0.69), and hematogenous metastasis (*P* = 0.07).

Cyclin D1 was localized to the nucleus in esophageal squamous cell carcinoma and was negative in normal esophageal mucosa or positive only in basal cells, as shown in Figures 1(i)–1(l). The positive expression rate of cyclin D1 in esophageal squamous cell carcinoma patients was 41.8% (72/172). Cyclin D1-positive expression was significantly associated with tumor size (*P* = 0.02, *P* < 0.05). There was no significant relationship between cyclin D1 and other clinicopathological parameters (*P* > 0.05) (Table 3).

### 3.3. Correlation of MACC1, c-Met, and Cyclin D1 Protein Expression

Spearman's rank correlation analysis was performed on 172 cases of esophageal squamous cell carcinoma. There was a significant positive correlation between the expression of MACC1 and c-Met (*R* = 0.485, *P* < 0.001). The expression of MACC1 was positively correlated with cyclin D1 (*R* = 0.177; *P* = 0.02). There was also a significant positive correlation between the expression of c-Met and cyclin D1 (*R* = 0.261; *P* = 0.001), as shown in Tables 4 and 5. These three proteins are highly expressed in esophageal squamous cell carcinoma. Based on their correlation, it is speculated that the MACC1/c-Met/cyclin D1 axis may promote the development of ESCC.

### 3.4. Prognostic Factors for OS and PFS

**Figure 2:**
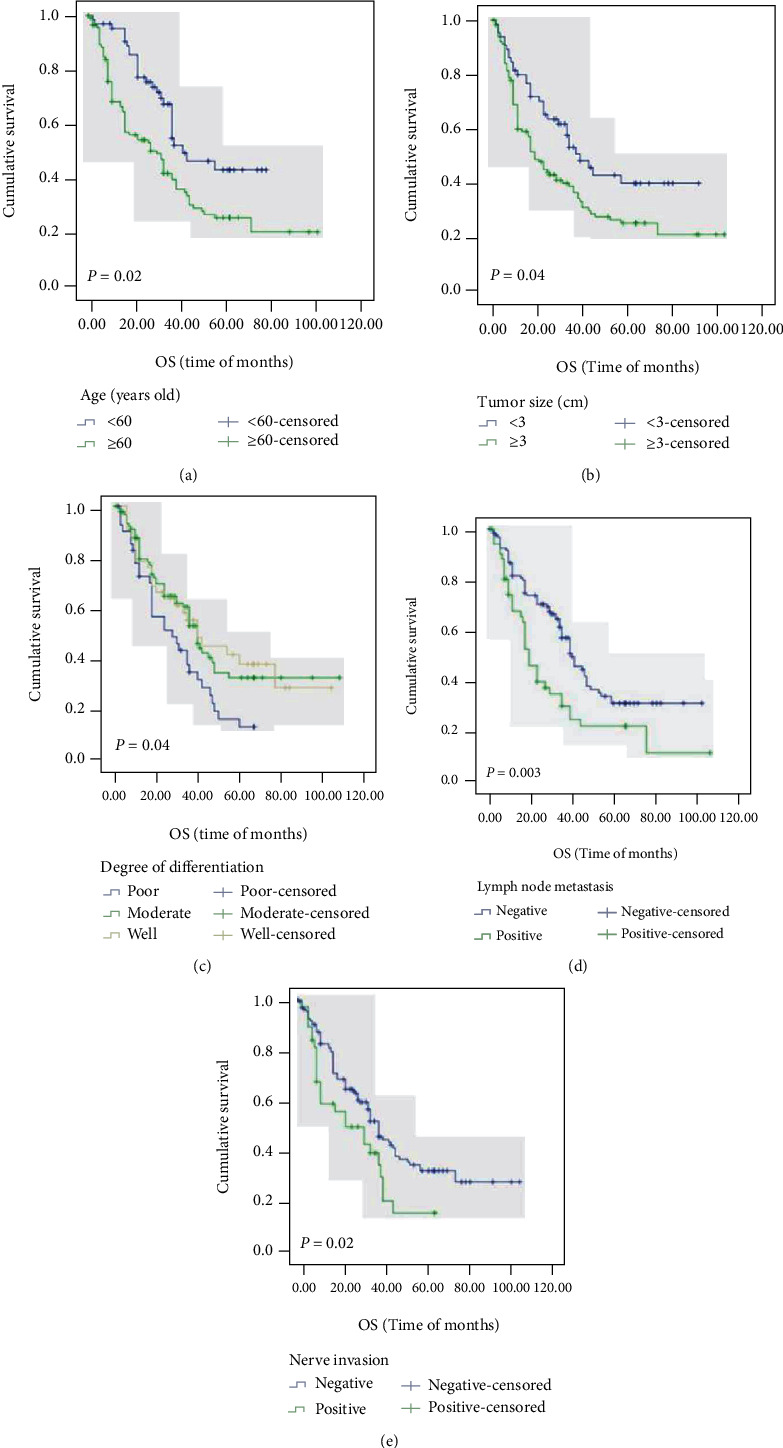
OS analysis of patients' clinicopathological parameters with ESCC using the Kaplan–Meier method. Notes: OS according to (a) age (*P* = 0.02), (b) tumor size (*P* = 0.04), (c) degree of differentiation (*P* = 0.04), (d) lymph node metastasis (*P* = 0.003), and (e) nerve invasion (*P* = 0.02). Abbreviations: OS: overall survival; PFS: progression-free survival; ESCC: esophageal squamous cell carcinoma.

**Figure 3:**
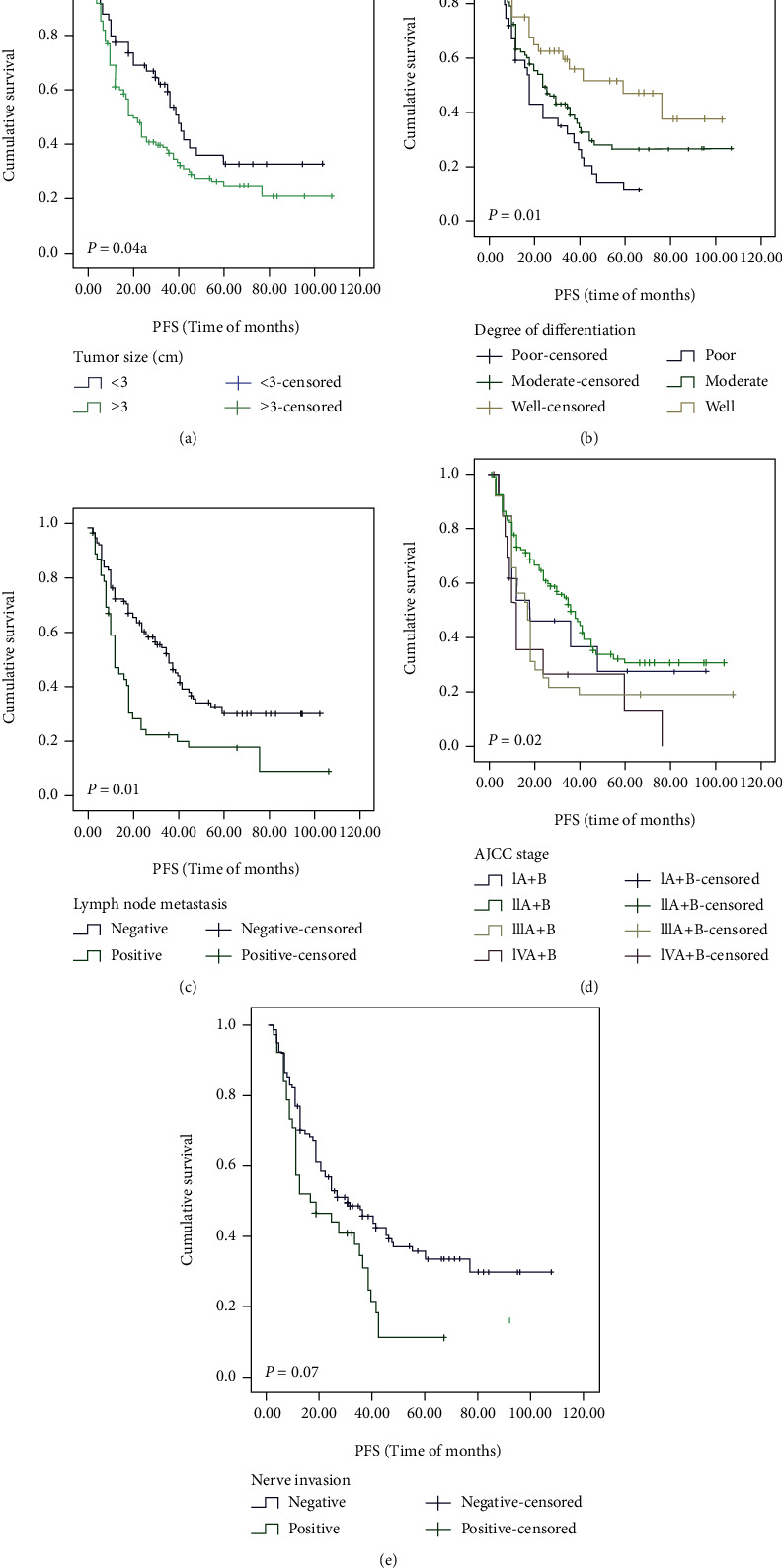
PFS analysis of patients' clinicopathological parameters with ESCC using the Kaplan–Meier method. Notes: PFS according to (a) tumor size (*P* = 0.04), (b) degree of differentiation (*P* = 0.01), (c) lymph node metastasis (*P* = 0.001), (d) AJCC stage (*P* = 0.02), and (e) nerve invasion (*P* = 0.007). Abbreviations: OS: overall survival; PFS: progression-free survival; ESCC: esophageal squamous cell carcinoma.

**Figure 4:**
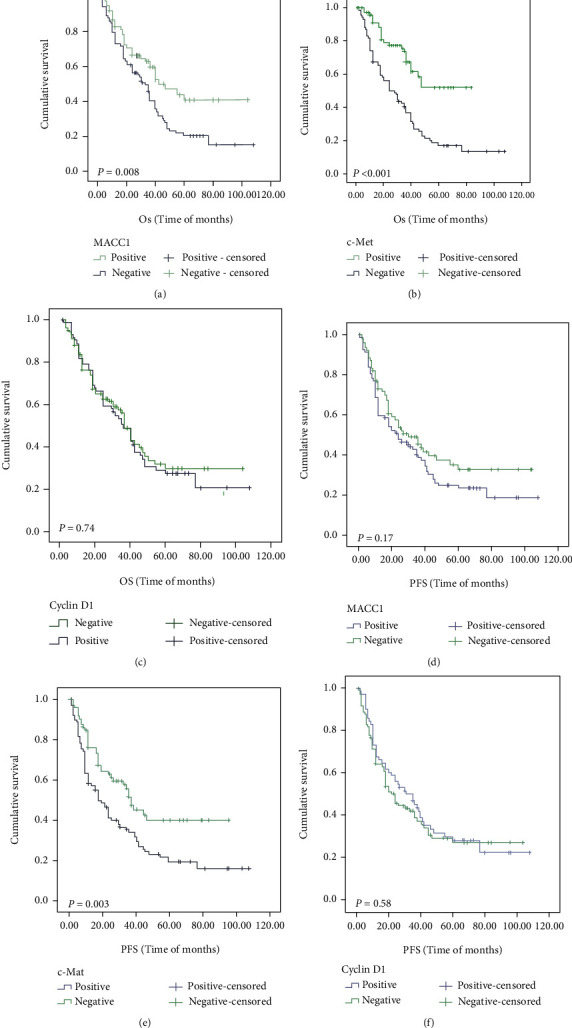
OS and PFS analysis of patients MACC1, c-Met, and cyclin D1 expression with ESCC using the Kaplan–Meier method. Notes: OS according to (a) MACC1 expression (*P* = 0.008), (b) c-Met expression (*P* < 0.001), and (c) cyclin D1 expression (*P* = 0.74). PFS according to (d) MACC1 expression (*P* = 0.17), (e) c-Met expression (*P* = 0.003), and (f) cyclin D1 expression (*P* = 0.58). Abbreviations: OS: overall survival; PFS: progression-free survival; ESCC: esophageal squamous cell carcinoma.

The Kaplan–Meier method was used to investigate the relationship between protein expression level and survival rate of esophageal squamous cell carcinoma patients. As shown in Table 6 and Figures 2 and 3, the K-M univariate analysis showed that the overall survival rate of ESCC patients was correlated with age (*P* = 0.02, Figure 2(a)), tumor size (*P* = 0.04, Figure 2(b)), degree of differentiation (*P* = 0.04, Figure 2(c)), lymph node metastasis (*P* = 0.003, Figure 2(d)), nerve invasion (*P* = 0.02, Figure 2(e)), etc. However, it was not correlated with gender, ethnicity, tumor location, invasion depth, AJCC stage, vascular invasion, and hematogenous metastasis. Tumor size (*P* = 0.04, Figure 3(a)), degree of differentiation (*P* = 0.01, Figure 3(b)), lymph node metastasis (*P* = 0.001, Figure 3(c)), AJCC stage (*P* = 0.02, Figure 3(d)), and nerve invasion (*P* = 0.007, Figure 3(e)) were associated with progression-free survival. As shown in Table 6 and Figure 4, patients with MACC1- (*P* = 0.008, Figure 4(a)) and c-Met- (*P* < 0.001, Figure 4(b)) positive expressions had poorer OS, and patients with high c-Met expression had worse PFS (*P* = 0.003, Figure 4(e)). However, cyclin D1 (*P* = 0.74, Figure 4(c)) expression was not associated with overall survival, and MACC1 (*P* = 0.17, Figure 4(d)) and cyclin D1 (*P* = 0.58, Figure 4(f)) expression was not related to progression-free survival.

In Table 7, the Cox multivariate regression analysis showed that positive MACC1 expression was associated with worse OS in patients (95% confidence interval (CI): 0.393-0.884, hazard ratio (HR): 0.589, *P* = 0.01), and positive c-Met expression was associated with worse OS (95% CI: 0.237-0.582, HR: 0.371, *P* < 0.001) and PFS (95% CI: 0.377-0.829, HR: 0.559, *P* = 0.004).

In summary, we analyzed the effects of various factors on overall survival and progression-free survival. The prognosis of patients with tumor size ≥ 3 cm was worse. Patients with high differentiation have poor prognosis, and patients with high differentiation have good prognosis. The survival time of patients with lymph node metastasis was significantly lower than that of patients without lymph node metastasis. Patients with nerve invasion have a poor prognosis. The overall survival rate of MACC1 and c-Met positive patients was significantly lower than that of negative patients.

### 3.5. The Expression of MACC1, c-Met, and Cyclin D1 Affected the Overall Survival Rate and Progression-Free Survival Time of Postoperative Chemotherapy Patients

In our study, 62 patients (36.0%) received postoperative chemotherapy. Among them, MACC1 was highly expressed in 29 cases (46.8%) and negative in 33 cases (53.2%). The positive rate of c-Met was 48.4%, with 30 cases positive and 32 cases negative. Cyclin D1 expression was high in 23 cases (37.1%) and negative in 39 cases (62.9%).

**Figure 5:**
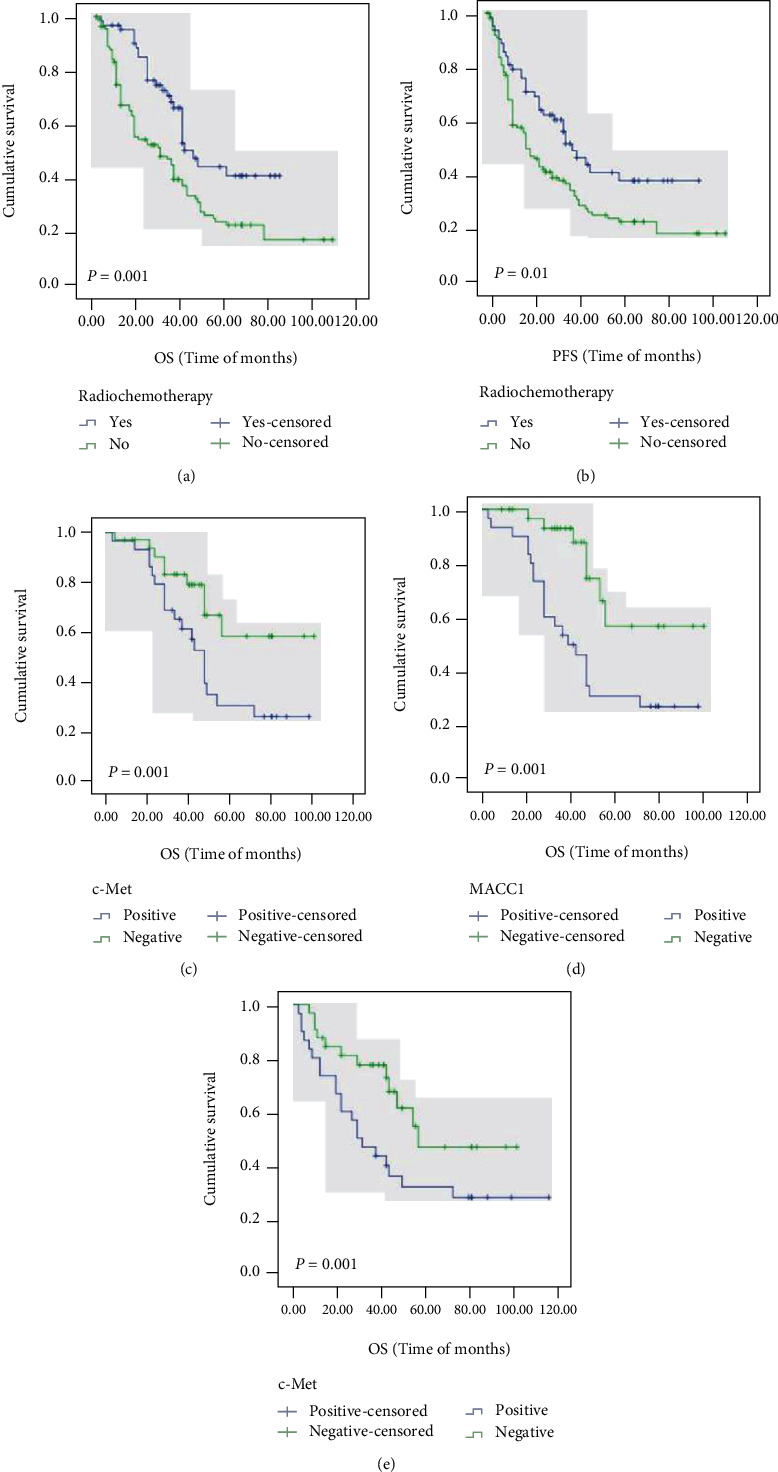
OS and PFS of ESCC patients after chemotherapy were analyzed by the Kaplan–Meier method. Notes: (a) OS according to postoperative chemoradiotherapy (*P* = 0.001); (b) PFS according to postoperative chemoradiotherapy (*P* = 0.01); (c) OS according to MACC1 expression (*P* = 0.03); (d) OS according to c-Met expression (*P* = 0.003); (e) PFS according to c-Met expression (*P* = 0.03). Abbreviations: OS: overall survival; PFS: progression-free survival; ESCC: esophageal squamous cell carcinoma.

The results of univariate analysis, as shown in Figure 5, showed that ESCC patients who received postoperative chemoradiotherapy had longer overall survival (*P* = 0.001, Figure 5(a)) and progression-free survival (*P* = 0.01, Figure 5(b)) than those who did not receive chemoradiotherapy. Further analysis showed that among the patients receiving chemoradiotherapy, patients with negative expression of MACC1 (*P* = 0.03, Figure 5(c)) and c-Met (*P* = 0.003, Figure 5(d)) survived longer. In progression-free survival, patients with positive c-Met expression (*P* = 0.03, Figure 5(e)) had a significantly lower survival rate than patients with negative c-Met expression.

These results suggest that MACC1 and c-Met may be independent factors affecting the poor prognosis of patients with esophageal cancer.

### 3.6. Effect of Coexpression of MACC1, c-Met, and Cyclin D1 on Prognosis of Patients with Esophageal Squamous Cell Carcinoma

As shown in Table 8, univariate survival analysis showed that MACC1, c-Met, and cyclin D1 were coexpressed (*P* < 0.001), MACC1 and c-Met were coexpressed (*P* < 0.001), and c-Met and cyclin D1 were coexpressed (*P* = 0.004). The effect on OS of patients with esophageal squamous cell carcinoma was statistically significant, but the coexpression of MACC1 and cyclin D1 (*P* = 0.08) had no effect on OS. However, further multivariate analysis showed that MACC1, c-Met, and cyclin D1 coexpression (*P* = 0.001), MACC1 and c-Met coexpression (*P* < 0.001), and c-Met and cyclin D1 coexpression (*P* = 0.003) had significant effects on OS influence. The coexpression of MACC1 and c-Met (*P* = 0.04) had an effect on the PFS of patients. Likewise, the multivariate analysis showed that MACC1 and c-Met coexpression (*P* = 0.001) was associated with PFS. The above results combined with the results in Tables 4, 6, and 7 infer that inhibiting the expression of both MACC1 and c-Met proteins at the same time is beneficial to prolong the overall survival and progression-free survival of patients with esophageal squamous cell carcinoma.

## 4. Discussion

In China, 90% of patients diagnosed with EC have advanced to the middle and late stage. Although surgery is the main means for the treatment of EC, the long-term effect is poor, the postoperative quality of life of patients is poor, and the overall 5-year survival rate is less than 20%. With the development of research, the application of molecular targeted drugs and immunotherapy has brought new hope for the treatment of patients with advanced EC, and it is of great significance to seek new molecular therapeutic targets for the adjuvant therapy of patients with esophageal cancer.

Hepatocyte growth factor (HGF) binds to the c-mesenchymal-epithelial transition (c-Met) receptor to activate the downstream signaling pathway, and it plays an important role in the occurrence and development of various cancers [22]. Tumor microenvironment is a key factor in tumor progression, and its expression is related to immune function of the body. Downstream immune-related genes activated by the HGF/c-Met pathway can regulate immune-related pathways, then affect the degree of immune cell infiltration, and thus affect the prognosis of tumor patients [23]. MACC1 promotes tumor development by regulating the HGF/c-Met pathway and microtubule stability [13] and is a key regulator of the c-Met pathway [13, 24, 25]. Cyclin D1 (CCND1) is an important cell cycle regulator that is considered a downstream target of c-Met [26]. In hepatocellular carcinoma cell lines, c-Met enhanced FAK activation in a FAK kinase-dependent manner and coinduced the activation of the AKT/ERK/cyclin D1 signaling pathway, thereby upregulating cyclin D1 expression and inducing tumor cell proliferation [27]. The purpose of this study was to analyze the expression of MACC1, c-Met, and cyclin D1 in esophageal squamous cell carcinoma and their relationship with clinicopathological parameters and to initially clarify the mechanism of action of the three in esophageal squamous cell carcinoma.

Metastasis-associated in colon cancer-1 (MACC1) is a novel prognostic, predictive, and causal biomarker for tumor progression and metastasis in many cancer types. Imbastari et al. found that MACC1 plays a role in endocytosis by regulating transmembrane receptor uptake and circulation, leading to intracellular upregulation of MACC1 expression. Increased MACC1 expression also leads to EGFR and its mediated downstream signaling pathway activation and cell proliferation, promoting the progression of colorectal cancer [28]. Cheng et al. found that overexpression of MACC1 was significantly associated with 5-year overall survival, metastasis-free survival, and disease-free survival (*P* < 0.05) and was significantly associated with recurrence-free survival (*P* > 0.05), the expression of MACC1 was significantly correlated with the expression of vimentin and E-cadherin, and MACC1 promoted the progression of nasopharyngeal carcinoma through the EMT process [29]. MACC1 can bind to the c-Met promoter and enhance the proliferation of osteosarcoma cells and endothelial cells through the HGF/c-Met signaling pathway. It also promotes angiogenesis by regulating microtubule dynamics, thereby promoting OS progression [13]. These results suggest that MACC1 can be used as a new molecular therapeutic target and prognostic marker.

In our study, the positive expression rate of MACC1 in esophageal squamous cell carcinoma was 53.5%. Analysis of clinicopathological parameters of ESCC patients showed that MACC1-positive expression was associated with tumor size (*P* = 0.02). In Kazakh patients, MACC1-positive expression was associated with tumor size (*P* = 0.03) and tumor invasion depth (*P* = 0.02). In Han nationality patients, MACC1-positive expression was associated with nerve invasion (*P* = 0.03). The K-M survival analysis showed that the overall survival of patients with positive MACC1 expression was significantly shorter than that of patients with negative MACC1 expression (*P* = 0.008), which was also validated by the Cox regression analysis (*P* = 0.01). Our results suggest that MACC1 is a cancer-promoting factor in patients with esophageal squamous cell carcinoma, and its high expression indicates poor prognosis in patients.

The c-Met tyrosine kinase plays an important role in human cancers. Armstrong et al. used immunohistochemical methods to find persistently high c-Met expression in colorectal cancer, and patients with high c-Met expression had poor prognosis [30]. Yang et al. found that the positive rate of c-Met in gastric cancer was 24.8%. c-Met expression was positively correlated with PDL1 expression. c-Met regulates PDL1 expression through an AKT-dependent pathway. Positive expression of c-Met plays an important prognostic role in disease-free survival (*P* = 0.03) [31]. Zhao et al. found that c-Met inhibition increased the secretion of various cytokines, including CCL2, IL8, or leukemia suppressor, and promoted the interaction between these cytokine receptors and Janus kinase 1/2 (JAK1/2), thus activating the JAKs/STAT3 signaling pathway and thus inhibiting the progression of esophageal squamous cell carcinoma [32]. For example, the c-Met inhibitor INC280 inhibits the enhancement of phosphorylated Met (p-Met) protein expression, and c-Met inhibitors inhibit pancreatic cancer metastasis in liver metastases mouse models of c-Met overexpressed cells [33]. ABN401 is a novel synthetic c-Met inhibitor that enhances the efficacy of non-small-cell lung cancer (NSCLC) by inhibiting c-Met expression in tumor cells and the activation of related signaling pathways [34]. These results suggest that c-Met plays an important role in tumor progression, and c-Met inhibitors can be used as adjuvant therapy for malignant tumors.

Our current results showed that 98 of 172 patients with esophageal squamous cell carcinoma had positive c-Met expression, accounting for 57.0%. Correlation analysis results are as follows: the positive expression of c-Met was significantly associated with ethnic group (*P* = 0.01), which was because esophageal squamous cell carcinoma has obvious regional characteristics, with a high incidence in Xinjiang region, and Kazakh people in Xinjiang region have bad daily eating habits (like eating hot food and raw food) [35]. Univariate survival analysis found that overall survival (*P* < 0.001) and progression-free survival (*P* = 0.003) were significantly shorter. The Cox risk ratio model suggested that c-Met might be a marker for poor prognosis of esophageal cancer. This is consistent with previous studies. c-Met can be used as a prognostic marker in patients with esophageal squamous cell carcinoma. Patients with positive c-Met expression have a worse prognosis, and related c-Met inhibitors can improve the efficacy.

Cyclin D1, an important regulator of cell cycle, carries out a central role in the pathogenesis of cancer determining uncontrolled cellular proliferation. Zhao et al. found that GDF-5 promoted epidermal stem cell proliferation in mice through the Foxg1 cyclin D1 signaling pathway [36]. In melanoma, Kaufmann et al. found increased expression of cyclin D1 in the aggressive thin melanin group compared with in situ melanoma [37]. Multiple meta-analyses suggest that cyclin D1 is the most important source of scientific evidence to study its prognostic value in human cancer. For example, immunohistochemical assessment of cyclin D1 overexpression can be used as a prognostic biomarker in OSCC [38]. Furthermore, cyclin D1 amplification was significantly associated with clinicopathological variables in breast cancer patients and could be used as an indicator of poor prognosis in breast cancer patients [39]. Another study found that cyclin D1 was an unfavorable prognostic factor in colorectal cancer patients [40]. However, studies have also shown that cyclin D1 is a predictor of good prognosis in patients with urologic cancers, such as renal cell carcinoma [41] and bladder cancer [42]. These findings suggest that cyclin D1 plays a key role in cell proliferation and tumor progression and that cyclin D1 can be used as a prognostic marker in clinical practice.

Immunohistochemical methods showed that cyclin D1 was expressed in ESCC tissue and normal esophageal mucosa adjacent to cancer. In normal esophageal mucosa, it was expressed in the basal layer and was positive in the nucleus; it was highly expressed in ESCC. Cyclin D1's high expression was correlated with tumor size (*P* = 0.02), but not with age, gender, ethnicity, tumor location, differentiation, lymph node metastasis, invasion depth, AJCC stage, vascular invasion, nerve invasion, and blood-derived metastasis (*P* > 0.05). Survival analysis showed that cyclin D1's high expression was not associated with overall survival (*P* = 0.74) and progression-free survival (*P* = 0.58) of esophageal squamous cell carcinoma patients. The coexpression of MACC1, c-Met, and cyclin D1 has an effect on the survival of ESCC patients, and the coexpression of MACC1 and cyclin D1 and the coexpression of c-Met and cyclin D1 have a significantly poor prognosis (*P* < 0.05). The results indicated that the positive expression of cyclin D1 promoted the proliferation of ESCC. Although the expression of cyclin D1 alone was not associated with ESCC prognosis in our study, our results suggest that cyclin D1 may be a key factor in the MACC1/c-Met signaling pathway, and the combined expression with MACC1 and c-Met may serve as a new esophageal research idea for prognosis of patients with squamous cell carcinoma. The reason why the results of previous studies were not suppressed may be related to the sample size and the deviation of patient information during the follow-up period, so it is necessary to expand the sample size for further analysis.

**Figure 6:**
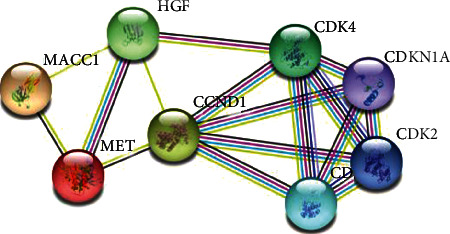
Connection diagram of MACC1, c-Met, and cyclin D1.

In previous studies, the HGF/c-Met signaling pathway is a key factor in tumor progression [8, 22]. We found that MACC1 is an important activator of c-Met. MACC1 binds to the positive feedback of the c-Met promoter region to activate the HGF/c-Met pathway, upregulates the expression of downstream target cyclin D1, and promotes tumor cell proliferation, invasion, metastasis, and epithelial mesenchymal transformation (EMT) [11, 26, 34]. The STRING database query showed that MACC1, c-Met, and cyclin D1 were coexpressed in human cells (Figure 6). Spearman's correlation analysis showed that MACC1 expression was positively correlated with c-Met expression (*R* = 0.485, *P* < 0.001), c-Met expression was positively correlated with cyclin D1 expression (*R* = 0.261, *P* = 0.001), and MACC1 expression was positively correlated with c-Met expression (*R* = 0.177, *P* = 0.02). Our results suggest that the MACC1/c-Met/cyclin D1 axis may play a role in promoting the development of ESCC and influencing the prognosis of ESCC.

Our further analysis found that the coexpression of three proteins, MACC1, c-Met, and cyclin D1, had an impact on the overall survival of ESCC, which again fully demonstrated that MACC1, c-Met, and cyclin D1 were jointly involved in the occurrence and development of ESCC and had an impact on the patient's survival prognosis. Therefore, we infer that inhibiting the expression of both MACC1 and c-Met protein simultaneously is beneficial to prolong the overall survival of ESCC and that MACC1 and c-Met protein can be used as molecular markers for poor prognosis of ESCC.

This study has some limitations. Only the roles of MACC1, c-Met, and cyclin D1 in ESCC and their effects on prognosis have been preliminarily confirmed. Immunohistochemical analysis could not determine the mechanism of action. Further studies combined with tissue, cell, and animal model experiments are needed to reveal the underlying mechanisms.

## 5. Conclusions

MACC1 and c-Met proteins are closely related to the occurrence and development of esophageal squamous cell carcinoma. MACC1 may affect the prognosis of patients with esophageal squamous cell carcinoma by regulating the expression of the c-Met/cyclin D1 axis. New prognostic markers for cancer are needed. The above results provide a theoretical basis for new therapeutic targets for ESCC.

## Figures and Tables

**Table 1:** General characteristics of ESCC patients.

Clinical characteristics	*n* (%)
*Age (years)*	
<60	70 (40.7%)
≥60	102 (59.3%)
*Gender*	
Male	118 (68.6%)
Female	54 (31.4%)
*Ethnicity*	
Han	58 (33.7%)
Kazakh	114 (66.3%)
*Tumor location*	
Upper	6 (3.5%)
Middle	96 (55.8%)
Lower	70 (40.7%)
*Tumor size(cm)*	
<3	51 (29.7%)
≥3	121 (70.3%)
*Differentiation*	
Poor	61 (21.3%)
Moderate	123 (53.0%)
Well	48(20.7%)
*Lymph metastasis*	
Negative	121 (70.3%)
Positive	51 (29.7%)
*Invasive depth*	
Mucosa	5 (2.9%)
Muscularis	78 (45.3%)
Full thickness	89 (51.7%)
*AJCC stage*	
IA+B	13 (7.6%)
IIA+B	113 (65.7%)
IIIA+B	32 (18.6%)
IVA+B	14 (8.1%)
*Vascular invasion*	
Negative	141 (82%)
Positive	31 (18.0%)
*Nerve invasion*	
Negative	135 (78.5%)
Positive	37 (21.5%)
*Hematogenous metastasis*	
Negative	146 (84.9%)
Positive	26 (15.1%)
*Radiochemotherapy*	
Yes	62 (36.0%)
No	110 (64.0%)

Abbreviations: ESCC: esophageal squamous cell carcinoma; AJCC: American Joint Committee on Cancer.

**Table 2:** The expression of MACC1 and its relationship with clinicopathological parameters in Han and Kazakh patients of ESCC.

Clinical characteristics (Han vs. Kazakh)	Han		*P* value	Kazakh		*P* value
	Positive (*n* = 33)	Negative (*n* = 25)		Positive (*n* = 59)	Negative (*n* = 55)	
*Age*						
<60 (31.0% vs. 45.6%)	11 (19.0%)	7 (12.1%)		32 (28.1%)	20 (17.5%)	
≥60 (69.0% vs. 54.4%)	22 (37.9%)	18 (31.0%)	0.67	27 (23.7%)	35 (30.7%)	0.06
*Gender*						
Male (74.1% vs. 65.8%)	24 (41.4%)	19 (32.8%)		37 (32.5%)	38 (33.3%)	
Female (25.9% vs. 34.2%)	9 (15.5%)	6 (10.3%)	0.78	22 (19.3%)	17 (14.9%)	0.47
*Tumor location*						
Upper (6.9% vs. 1.8%)	3 (5.2%)	1 (1.7%)		1 (0.9%)	1 (0.9%)	
Middle (48.3% vs. 59.6%)	15 (25.9%)	13 (22.4%)		33 (28.9%)	35 (30.7%)	
Lower (44.8% vs. 38.6%)	15 (25.9%)	11 (19.0%)	0.71	25 (21.9%)	19 (16.7%)	0.69
*Tumor size*						
<3 cm (29.3% vs. 29.8%)	6 (10.3%)	11 (19.0%)		14 (12.3%)	20 (17.5%)	
≥3 cm (70.7% vs. 70.2%)	27 (46.6%)	14 (24.1%)	0.03	45 (39.5%)	35 (30.7%)	0.14
*Degree of differentiation*						
Poor (34.5% vs. 18.4%)	12 (20.7%)	8 (13.8%)		13 (11.4%)	8 (7.0%)	
Moderate (53.4% vs. 51.8%)	16 (27.6%)	15 (25.9%)		25 (21.9%)	34 (29.8%)	
Well (12.1% vs. 29.8%)	5 (8.6%)	2 (3.4%)	0.60	21 (18.4%)	13 (11.4%)	0.12
*Lymph node metastasis*						
Negative (63.8% vs. 73.7%)	22 (37.9%)	15 (25.9%)		44 (38.6%)	40 (35.1%)	
Positive (36.2% vs. 26.3%)	11 (19.0%)	10 (17.2%)	0.60	15 (13.2%)	15 (13.2%)	0.82
*Invasive depth*						
Mucosa (6.9% vs. 0.9%)	2 (3.4%)	2 (3.4%)		1 (0.9%)	0 (0.0%)	
Muscularis (48.3% vs. 43.9%)	11 (19.0%)	17 (29.3%)		27 (23.7%)	23 (20.2%)	
Full thickness (44.8% vs. 55.3%)	20 (34.5%)	6 (10.3%)	0.02	31 (27.2%)	32 (28.1%)	0.45
*AJCC stage*						
IA+B (6.9% vs. 7.9%)	3 (5.2%)	1 (1.7%)		8 (7.0%)	1 (0.9%)	
IIA+B (33.3% vs. 65.8%)	20 (34.5%)	18 (31.0%)		36 (31.6%)	39 (34.2%)	
IIIA+B (20.7% vs. 17.5%)	7 (12.1%)	5 (8.6%)		11 (9.6%)	9 (7.9%)	
IVA+B (6.9% vs. 5.8%)	3 (5.2%)	1 (1.7%)	0.70	4 (3.5%)	6 (5.3%)	0.11
*Vascular invasion*						
Negative (82.8% vs. 81.6%)	27 (46.6%)	21 (36.2%)		49 (43.0%)	44 (38.6%)	
Positive (17.2% vs. 18.4%)	6 (10.3%)	4 (6.9%)	0.83	10 (8.8%)	11 (9.6%)	0.68
*Nerve invasion*						
Negative (82.8% vs. 76.3%)	27 (46.6%)	21 (36.2%)		50 (43.9%)	37 (32.5%)	
Positive (17.2% vs. 23.7%)	6 (10.3%)	4 (6.9%)	0.83	9 (7.9%)	18 (15.8%)	0.03
*Hematogenous metastasis*						
Negative (81.0% vs. 86.8%)	27 (46.6%)	20 (34.5%)		50 (43.9%)	49 (43.0%)	
Positive (19.0% vs. 13.2%)	6 (10.3%)	5 (8.6%)	0.87	9 (7.9%)	6 (5.3%)	0.49

Abbreviations: ESCC: esophageal squamous cell carcinoma; AJCC: American Joint Committee on Cancer.

**Table 3:** Correlation of MACC1, c-Met, and cyclin D1 expression with clinicopathological features in 172 ESCC patients.

	Total		*P* value	Total		*P* value	Total		*P* value
	MACC1			c-Met			Cyclin D1		
	Positive 92 (53.5%)	Negative 80 (46.5%)		Positive 98 (57.0%)	Negative 74 (43.0%)		Positive 72 (41.9%)	Negative 100 (58.1%)	
*Age*									
<60 (*n* = 70, 40.7%)	43 (25.0%)	27 (15.7%)		45 (26.2%)	25 (14.5%)		35 (20.3%)	35 (20.3%)	
≥60 (*n* = 102, 59.3%)	49 (28.5%)	53 (30.8%)	0.08	53 (30.8%)	49 (28.5%)	0.11	37 (21.5%)	65 (37.8%)	0.07
*Gender*									
Male (*n* = 118, 68.6%)	61 (35.5%)	57 (33.1%)		66 (38.4%)	52 (30.2%)		48 (27.9%)	70 (40.7%)	
Female (*n* = 54, 31.4%)	31 (18.0%)	23 (13.4%)	0.47	32 (18.6%)	22 (12.8%)	0.68	24 (14.0%)	30 (17.4%)	0.64
*Ethnicity*									
Han (*n* = 58, 33.7%)	33 (19.2%)	25 (14.5%)		41 (23.8%)	17 (9.9%)		23 (13.4%)	35 (20.3%)	
Kazakh (*n* = 114, 66.3%)	59 (34.3%)	55 (32.0%)	0.52	57 (33.1%)	57 (33.1%)	0.01	49 (28.5%)	65 (37.8%)	0.16
*Tumor location*									
Upper (*n* = 6, 3.5%)	4 (2.3%)	2 (1.2%)		4 (2.3%)	2 (1.2%)		3 (1.7%)	3 (1.7%)	
Middle (*n* = 96, 55.8%)	48 (27.9%)	48 (27.9%)		51 (29.7%)	45 (26.2%)		40 (23.3%)	56 (32.6%)	
Lower (*n* = 70, 40.7%)	40 (23.3%)	30 (17.4%)	0.53	43 (25.0%)	27 (15.7%)	0.50	29 (16.9%)	41 (23.8%)	0.92
*Tumor size*									
<3 cm (*n* = 51, 29.7%)	20 (11.6%)	31 (18.0%)		25 (14.5%)	26 (15.1%)		28 (16.3%)	23 (13.4%)	
≥3 cm (*n* = 121, 70.3%)	72 (41.9%)	49 (28.5%)	0.02	73 (42.4%)	48 (27.9%)	0.17	44 (25.6%)	77 (44.8%)	0.02
*Degree of differentiation*									
Poor (*n* = 41, 23.8%)	25 (14.5%)	16 (9.3%)		25 (14.5%)	16 (9.3%)		16 (9.3%)	25 (14.5%)	
Moderate (*n* = 90, 52.4%)	41 (23.8%)	49 (28.5%)		45 (26.2%)	45 (26.2%)		38 (22.1%)	52 (30.2%)	
Well (*n* = 41, 23.8%)	26 (15.1%)	15 (8.7%)	0.09	28 (16.3%)	13 (7.6%)	0.12	18 (10.5%)	23 (13.4%)	0.90
*Lymph node metastasis*									
Negative (*n* = 121, 70.3%)	66 (38.4%)	55 (32.0%)		69 (40.1%)	52 (30.2%)		54 (31.4%)	67 (39.0%)	
Positive (*n* = 51, 29.7%)	26 (15.1%)	25 (14.5%)	0.67	29 (16.9%)	22 (12.8%)	0.98	18 (10.5%)	33 (19.2%)	0.26
*Invasive depth*									
Mucosa (*n* = 5, 2.9%)	3 (1.7%)	2 (1.3%)		3 (1.7%)	2 (1.2%)		1 (0.6%)	4 (2.3%)	
Muscularis (*n* = 78, 45.3%)	38 (22.0%)	40 (23.3%)		46 (26.7%)	32 (18.6%)		33 (19.2%)	45 (26.2%)	
Full thickness (*n* = 89, 51.8%)	51 (29.7%)	38 (22.0%)	0.52	49 (28.5%)	40 (23.3%)	0.87	38 (22.1%)	51 (29.7%)	0.58
*AJCC stage*									
IA+B (*n* = 13, 7.6%)	11 (6.4%)	2 (1.2%)		10 (5.8%)	3 (1.7%)		6 (3.5%)	7 (4.1%)	
IIA+B (*n* = 113, 65.7%)	56 (32.6%)	57 (33.1%)		59 (34.3%)	54 (31.4%)		48 (27.9%)	65 (37.8%)	
IIIA+B (*n* = 32, 18.6%)	18 (10.5%)	14 (8.1%)		21 (12.2%)	11 (6.4%)		12 (7.0%)	20 (11.6%)	
IVA+B (*n* = 14, 8.2%)	7 (4.1%)	7 (4.1%)	0.12	8 (4.7%)	6 (3.5%)	0.25	6 (3.5%)	8 (4.7%)	0.95
*Vascular invasion*									
Negative (*n* = 141, 82.0%)	76 (44.2%)	65 (37.8%)		79 (45.9%)	62 (36.0%)		59 (34.3%)	82 (47.7%)	
Positive (*n* = 31, 18.0%)	16 (9.3%)	15 (8.7%)	0.82	19 (11.0%)	12 (7.0%)	0.59	13 (7.6%)	18 (10.5%)	0.99
*Nerve invasion*									
Negative (*n* = 135, 78.5%)	77 (44.8%)	58 (33.7%)		78 (45.3%)	57 (33.1%)		55 (32.0%)	80 (46.5%)	
Positive (*n* = 37, 21.5%)	15 (8.7%)	22 (12.8%)	0.08	20 (11.6%)	17 (9.9%)	0.69	17 (9.9%)	20 (11.6%)	0.57
*Hematogenous metastasis*									
Negative (*n* = 146, 84.9%)	77 (44.8%)	69 (40.1%)		79 (45.9%)	67 (39.0%)		58 (33.7%)	88 (51.2%)	
Positive (*n* = 26, 15.1%)	15 (8.7%)	11 (6.4%)	0.64	19 (11.0%)	7 (4.1%)	0.07	14 (8.1%)	12 (7.0%)	0.18

Abbreviations: ESCC: esophageal squamous cell carcinoma; AJCC: American Joint Committee on Cancer.

**Table 4:** Correlation between MACC1, c-Met, and cyclin D1.

	MACC1 expression	
	*R*	*P* value
c-Met expression	0.485	<0.001
Cyclin D1 expression	0.177	0.02

**Table 5:** Correlation between c-Met and cyclin D1.

	Cyclin D1 expression	
	*R*	*P* value
c-Met expression	0.261	0.001

**Table 6:** Univariate analysis of factors associated with OS and PFS in ESCC patients.

Characteristic	OS			PFS		
	95% CI	*χ* ^2^	*P* value	95% CI	*χ* ^2^	*P* value
*Age (years)*						
<60/≥60	24.681-37.319 vs. 31.774-48.226	5.360	0.02	13.920-34.080 vs. 14.944-45.056	3.761	0.05
*Gender*						
Male/female	30.714-26.587 vs. 26.587-45.413	0.081	0.78	16.493-31.507 vs. 13.258-48.742	1.077	0.30
*Ethnicity*						
Han/Kazakh	25.206-46.794 vs. 33.988-46.012	0.164	0.67	16.037-31.963 vs. 19.773-42.227	1.136	0.29
*Tumor location*						
Upper/middle/lower	20.088-75.912 vs. 34.733-45.267 vs. 25.607 vs. 40.393	2.183	0.34	0-99.530 vs. 11.042-40.958 vs. 10.175 vs. 37.825	1.063	0.59
*Tumor size*						
<3 cm/≥3 cm	35.063-48.937 vs. 24.025-41.975	4.267	0.04	33.675-46.325 vs. 15.498-24.502	3.908	0.04
*Degree of differentiation*						
Poor/moderate/well	12.573-43.427 vs. 34.313-45.687 vs. 28.959-51.041	6.521	0.04	15.678-20.322 vs. 15.539-32.641 vs. 20.402-99.598	9.072	0.01
*Lymph node metastasis*						
Negative/positive	33.142-48.858 vs. 15.820-24.180	8.730	0.003	29.674-42.326 vs. 6.921-17.079	11.888	0.001
*Invasive depth*						
Mucosa/muscularis/full thickness	0-85.4 vs. 35.377-44.623 vs. 28.313-43.687	0.487	0.78	0-39.4 vs. 17.492-42.508 vs. 16.086-31.914	2.109	0.35
*AJCC stage*						
I/II/III/IV	6.073-65.927 vs. 33.591-48.409 vs. 17.713-22.287 vs. 5.721-54.279	7.261	0.06	0-46.624 vs. 28.118-43.882 vs. 12.85-21.15 vs. 8.911-15.089	9.911	0.02
*Vascular invasion*						
Negative/positive	30.609-41.391 vs. 31.516-48.484	0.274	0.60	15.312-38.688 vs. 11.752-36.248	0.634	0.43
*Nerve invasion*						
Negative/positive	34.737-45.263 vs. 6.270-41.730	5.197	0.02	19.232-40.768 vs. 0-32.479	7.272	0.007
*Hematogenous metastasis*						
Negative/positive	29.139-42.861 vs. 31.769-40.231	0.547	0.46	19.595-40.405 vs. 3.368-36.632	3.407	0.07
*MACC1 expression*						
Negative/positive	27.368-38.632 vs. 25.557-58.443	6.985	0.008	13.556-34.444 vs. 17.052-42.948	1.921	0.17
*c-Met expression*						
Negative/positive	29.822-43.453 vs. 49.128-65.143	21.173	<0.001	12.271-23.729 vs. 28.620-47.740	8.991	0.003
*Cyclin D1 expression*						
Negative/positive	27.638-44.362 vs. 31.777-40.223	0.111	0.74	21.643-48.357 vs. 18.494-29.506	0.302	0.58

Abbreviations: ESCC: esophageal squamous cell carcinoma; AJCC: American Joint Committee on Cancer; OS: overall survival; PFS: progression-free survival.

**Table 7:** Multivariate analysis of factors associated with OS and PFS for ESCC.

	OS			PFS		
	95% CI	HR	*P* value	95% CI	HR	*P* value
*Age (years)*						
<60/≥60	1.058-2.290	1.566	0.03	—	—	—
*Tumor size*						
<3 cm/≥3 cm	0.403-0.990	0.631	0.04	0.428-1.008	0.657	0.06
*Degree of differentiation*						
Poor/moderate/well	0.563-0.998	0.746	0.04	0.511-0.875	0.668	0.003
*Lymph node metastasis*						
Negative/positive	1.200-2.685	1.795	0.004	1.307-2.830	1.923	0.001
*AJCC stage*						
I/II/III/IV	—	—	—	1.076-1.795	1.39	0.01
*Nerve invasion*						
Negative/positive	1.058-2.586	1.654	0.03	1.147-2.638	1.739	0.009
*MACC1 expression*						
Negative/positive	0.393-0.884	0.589	0.01	—	—	—
*c-Met expression*						
Negative/positive	0.237-0.582	0.371	<0.001	0.377-0.829	0.559	0.004

Abbreviations: ESCC: esophageal squamous cell carcinoma; AJCC: American Joint Committee on Cancer; OS: overall survival; PFS: progression-free survival.

**Table 8:** Effects of coexpression of MACC1, c-Met, and cyclin D1 on the prognosis of ESCC.

Coexpression	OS		OS		PFS		PFS	
	Univariate		Multivariate		Univariate		Multivariate	
	Log-rank (Mantel-Cox)	*P* value	95% CI	*P* value	Log-rank (Mantel-Cox)	*P* value	95% CI	*P* value
MACC1, c-Met, and cyclin D1								
(+++ vs. ++- vs. +-- vs. ---)	17.946	<0.001	0.605-0.877	0.001	0.449	0.93	—	—
MACC1 and c-Met								
(++ vs. +- vs. --)	18.438	<0.001	0.456-0.767	<0.001	6.638	0.04	0.600-0.941	0.01
MACC1 and cyclin D1								
(++ vs. +- vs. --)	5.025	0.08	—	—	1.16	0.56	—	—
c-Met and cyclin D1								
(++ vs. +- vs. --)	10.932	0.004	0.534-0.878	0.003	0.216	0.90	—	—

Abbreviations: ESCC: esophageal squamous cell carcinoma; OS: overall survival; PFS: progression-free survival; 95% CI: 95% confidence interval.

## Data Availability

The datasets used and/or analyzed during the current study are available from the corresponding author on reasonable request. Requests for access to these data should be made to Yu-Qing Ma (yuqingm0928@126.com).
